# FOLFOX versus EOX as a neoadjuvant chemotherapy regimen for patients with advanced gastric cancer

**DOI:** 10.3892/etm.2013.1449

**Published:** 2013-12-13

**Authors:** WENJUN CHEN, JIANGUO SHEN, TAO PAN, WENXIAN HU, ZINONG JIANG, XIAOMING YUAN, LINBO WANG

**Affiliations:** 1 Department of Surgical Oncology, Sir Run Run Shaw Hospital, Zhejiang University College of Medicine, Hangzhou, Zhejiang 310016, P.R. China; 2 Department of Pathology, Sir Run Run Shaw Hospital, Zhejiang University College of Medicine, Hangzhou, Zhejiang 310016, P.R. China

**Keywords:** gastric cancer, neoadjunvant chemotherapy, oxaliplatin, epirubicin, capecitabine

## Abstract

Neoadjuvant chemotherapy is the preferred treatment of advanced gastric cancer. However, the choice of an optimal regimen remains controversial. The present study aimed to assess the effectiveness of preoperative chemotherapy with EOX and FOLFOX in Chinese patients with advanced gastric cancer. A total of 87 and 26 patients underwent FOLFOX and EOX regimens, respectively, for advanced gastric cancer between July 2004 and September 2012. Clinicopathological characteristics, pathological T stage, N stage and pathological response to tumour regression were retrospectively compared between the two groups. Following neoadjuvant chemotherapy, a higher number of patients manifested deeper invasive cancer in the FOLFOX group than those in the EOX group (P=0.047). In addition, a higher number of patients also exhibited metastatic lymph nodes in the FOLFOX group (67.8%) than in the EOX group (57.7%) (P=0.000). In the FOLFOX and EOX groups, 4 (4.6%) and 3 (11.5%) cases of complete regression were observed, respectively. A higher number of patients (38.5%) also exhibited tumour regression grades of 3 and 4 in the EOX group than in the FOLFOX group (19.5%) (P=0.047). Results of the present study suggest that the EOX regimen may be more effective than the FOLFOX regimen as preoperative chemotherapy for Chinese patients with advanced gastric cancer. The EOX regimen may be suitable for younger patients subjected to individual neoadjuvant chemotherapy.

## Introduction

Gastric cancer is considered the third and fifth most common cause of cancer-related mortalities in males and females worldwide, respectively. In addition, gastric cancer ranks fourth and fifth among the most common malignancies in males and females worldwide, respectively (1). Approximately two thirds of these cases occur in developing countries in Eastern Europe, South America and Asia. Specifically, 42% of the cases are recorded in China alone (2). Complete radical resection is important for the treatment of gastric cancer (3). Neoadjuvant chemotherapy is currently used worldwide as initial therapy for locally advanced gastric cancer, as this technique can increase the radical operation (R0) resection rate and improve overall survival (4). Neoadjuvant chemotherapy followed by surgery provides more benefits than postoperative chemotherapy (5). However, illness may be worsened in unresponsive cases and thus delay radical surgery. Suitable neoadjuvant chemotherapy is important to treat patients with advanced gastric cancer.

FOLFOX and ECF, including ECF-modified EOX are considered the main regimens in neoadjuvant chemotherapy. In China, FOLFOX regimen is commonly used due to its effectiveness and high compliance (6,7). In addition, the triplet regimen, EOX, may have a higher response rate than the doublet regimen, FOLFOX. However, the optimal approach in individual patients remains controversial due to the lack of well-established trials. Considering these findings, the present study retrospectively compared FOLFOX with EOX as neoadjuvant regimens in terms of the response rate and presence of side effects. This study aimed to investigate the optimal regimen that can be used for neoadjuvant chemotherapy.

## Subjects and methods

### Patients

A total of 655 patients with gastric cancer underwent surgery between July 2004 and September 2012 at the Department of Surgical Oncology, (Sir Run Run Shaw Hospital, Hangzhou, China). Among these patients, 87 and 26 patients underwent preoperative chemotherapy with FOLFOX and EOX regimens, respectively. The patients were recommended to undergo neoadjuvant chemotherapy if the following criteria were met: i) A pathological diagnosis of proven gastric carcinoma; ii) clinical stage of ≥T2 or lymph node metastasis; iii) Eastern Cooperative Oncology Group performance status of ≤2; iv) adequate organ function; and v) no active concomitant malignancy. The initial clinical evaluation was performed by upper gastrointestinal endoscopy and/or ultrasound endoscopy, abdominal computerised tomography (CT) with contrast and upper GI contrast. Radiological evaluations for staging were jointly reviewed by radiologists, surgeons and oncologists of Sir Run Run Shaw Hospital. Neoadjuvant treatment was administered according to the policy of The Tumor Centre of Sir Run Run Shaw Hospital. This treatment comprised chemotherapy or radiochemotherapy if the tumour was located around the gastro-oesophageal junction. Response to chemotherapy was evaluated following two courses according to the Response Evaluation Criteria in Solid Tumors (RECIST) criteria (8). In cases of partial response, two additional courses of chemotherapy were administered or the patients underwent surgery as soon as possible. This study was conducted in accordance with the declaration of Helsinki with approval from the ethics committee of Sir Run Run Shaw Hospital. Written informed consent was obtained from all participants.

### Surgery

Surgery was scheduled 1–2 weeks following neoadjuvant chemotherapy completion. After laparotomy, the extent of dissection and curative success of the procedure were determined. For the patients with curative resection, total or distal subtotal gastrectomy was performed depending on the location and macroscopic type of gastric cancer. Splenectomy was not routinely performed unless direct tumour invasion in the spleen was suspected or if accidental injury occurred during surgery. D2 or >D2 lymphadenectomy was performed according to the rules of the Japanese Research Society for Gastric Cancer (9).

### Neoadjuvant chemotherapy

Eighty-seven patients with locally advanced gastric cancer were treated with FOLFOX as follows: Intravenous (IV) administration of 85 mg/m^2^ oxaliplatin, 400 mg/m^2^ leucovorin and IV push administration of 400 mg/m^2^ fluorouracil on day 1, and 1,200 mg/m^2^ fluorouracil IV continuous infusion for 24 h on days 1 and 2. This regimen was repeated every 14 days. Twenty-six patients were treated with EOX according to the following protocol: IV administration of 50 mg/m^2^ epirubicin and 100 mg/m^2^ oxaliplatin on day 1, and 800 mg/m^2^ capecitabine *per os* twice a day on days 1–14. This regimen was repeated every 21 days.

### Assessment of pathological response

After dissection of the lymph nodes from the specimen, the stomach was placed on a flat board with the mucosal side up, pinned at the edges with stainless steel pins and fixed in a 10% buffered formalin solution. Tumor specimens of tumour were preserved in the paraffin block. Following surgery, all of the tumour specimens were examined by the pathologists. The pathological stage was determined according to the rules of the 7th edition of Union for International Cancer Control (UICC) and the stage grouping of the UICC/American Joint Committee on Cancer. The pathological response and histological tumour regression are considered as important predictors of survival in patients with gastric adenocarcinoma (10–12). Neoadjuvant treatment response was evaluated using tumour regression grade (TRG), which was microscopically evaluated using the scale proposed by Mandard *et al*(13). Regression was graded as follows: TRG 4, complete regression; TRG 3, isolated cell nests; TRG 2, more residual cancer cells but fibrosis still dominant; TRG 1, residual cancer outgrowing fibrosis; and TRG 0, absence of regressive changes. TRG 3 and 4 were defined as tumour regression for the purpose of statistical analysis based on previous studies (14). Our previous study also showed that patients with tumour regression exhibited higher overall survival than those without tumour regression (15).

The primary objective of this procedure was to determine non-inferiority in tumour regression of the triplet regimen EOX compared with that of the doublet regimen FOLFOX.

### Evaluation of neoadjuvant chemotherapy toxicity

Chemotherapy toxicity was graded according to National Cancer Institute standards (16). The indicators were bone marrow suppression, reaction of the gastrointestinal tract, hand-foot syndrome, peripheral neuropathy disorders and liver function.

### Statistical analysis

The primary objective of this analysis was to determine the inferiority of pathological tumour response in the triplet neoadjuvant chemotherapy EOX regimen compared with the doublet FOLFOX regimen. The side effects of the two regimens were also observed in patients with gastric cancer. The analysed clinicopathological variables included age, gender, clinical staging, tumour location, differentiation, size and chemotherapy cycles. Pretreated tumour size was determined by endoscopy, CT scan with contrast or both. For tumour differentiation, moderately- and well-differentiated tubular adenocarcinoma, papillary adenocarcinoma and mucinous adenocarcinoma of the well-differentiated subtypes were grouped as the differentiated type. Poorly-differentiated adenocarcinoma, signet ring cell carcinoma and mucinous adenocarcinoma of the poorly-differentiated subtype were grouped as the undifferentiated type. Statistical calculations were analysed using SPSS version 20.0 for Windows (SPSS Inc., Chicago, IL, USA) and Excel 2010 (Microsoft, Redmond, WA, USA). χ^2^ or Fisher’s exact tests were used to determine the significance of associations between pathological findings and categorical variables. P<0.05 was considered to indicate a statistically significant difference.

## Results

### Patient’s characteristics

A total of 113 patients undergoing neoadjuvant chemotherapy were enrolled in this study. Among these patients, 87 received FOLFOX and 26 received EOX. The study groups were well-balanced in terms of their baseline characteristics (Table I). Approximately four cycles of preoperative chemotherapy were scheduled in the two regimens. The patients in the EOX group were slightly younger than those in the FOLFOX group, but no significant difference in age was observed according to the χ^2^ test (P=0.073). CT scan with contrast showed the majority of patients presented metastatic lymph nodes prior to treatment. In particular, 56 (64.4%) and 18 (69.2%) cases exhibited positive lymph nodes in the FOLFOX and EOX groups, respectively. The malignant lesions located in the lower body of the stomach were present in more than half of the patients, 47 (54%) and 14 (53.8%) cases showed malignant lesions in the FOLFOX and EOX groups, respectively.

### Pathological response following neoadjuvant chemotherapy

According to TRG, 4 (4.6%) and 3 (11.5%) cases exhibited complete regression in the FOLFOX and the EOX groups, respectively. A significantly higher number of patients (38.5%) with tumour regression (TRG 3 and 4) were found in the EOX group compared with the FOLFOX group (19.5%) (P=0.047).

### Differences in radical gastrectomy and pathological T and N stages following neoadjuvant chemotherapy

In the patients subjected to the EOX regimen, ~84.6% showed R0 resection and this percentage was higher than that of the patients subjected to the FOLFOX regimen (79.3%). According to results of the CT scan with contrast, the pretreated clinical T and N stages were similar in the FOLFOX and EOX groups. Following neoadjuvant chemotherapy, the number of patients with metastatic lymph nodes in the FOLFOX group (67.8%) was significantly higher than that in the EOX group (57.7%) (P=0.000). In addition, the number of patients with deeper invasive cancer was significantly higher in the FOLFOX group compared with that of the EOX group (P=0.047; Table II).

### Safety and quality of life

Table III shows the incidence of adverse events in the two groups. No lethal incident was identified in all of the patients. The total vomiting and nausea in grades III and IV were significantly lower in the FOLFOX group (20.7%) compared to the EOX group (30.7%) (P=0.029). Three patients (11.5%) vomited >10 times in 24 h and the remaining patients received parenteral support. All of the patients recovered 3 or 4 days following infusion of the chemotherapy agents. Grade III neutropenia was more frequent in the EOX group (11.5%) than in the FOLFOX group (4.6%), but this difference was not significant (P=0.635). Grade I hand-foot syndrome occurred at a significantly higher rate in the EOX group (34.6%) than in the FOLFOX group (16.1%) (P=0.040), indicating that this syndrome was mild. Additional adverse events were not severe in the two groups prior to surgery (Table III).

## Discussion

Despite a gradual decline in the overall incidence worldwide, gastric cancer remains the second most common cause of cancer-related mortalities. Nearly two thirds of gastric cancer cases occur in developing countries in Eastern Europe, South America and Asia, where 42% of the cases are recorded in China alone. In an early stage locoregionally confined disease, the five-year survival rate rarely exceeds 25–35% (17). According to the National Cancer Database (18), 10.7 and 39.2% of the patients manifest early stage disease (stage 0-IA) and metastatic disease (stage IV), respectively. Therefore, the most prevalent subgroup at 50.2% consisted of patients diagnosed with locally advanced but resectable disease (stage IB-III). This group of patients also shows a potentially curable disease but exhibits the highest risk of recurrence and metastasis. Thus, the lower number of patients with early stage disease in China indicates that patients with advanced disease require adjuvant therapy since surgery is the primary treatment for early stage gastric cancer. The results of two Phase III randomised trials, namely MAGIC (4) and FNCLCC/FFCD (19), have markedly suggested that neoadjuvant chemotherapy is beneficial. The advantages of neoadjuvant chemotherapy include a decrease in the stage of disease severity, a higher rate of R0 resection, tumour regression and avoidance of unnecessary surgery (4,20,21).

Radical surgery is the main method used to treat early stage gastric cancer. The five-year survival rate can increase from 85 to 95% following R0 surgery. However, the five-year survival rate of advanced gastric cancer cases is <50% (22). Thus, a suitable regimen of preoperative chemotherapy is important for advanced gastric carcinoma. Surgeons and patients require a more effective and less toxic regimen to downstage the disease.

For gastrointestinal malignant tumours, fluorouracil and cisplatin are used as basic chemotherapy agents. The FOLFOX chemotherapy regimen has been established as the standard colorectal cancer chemotherapy. This regimen is highly effective and well tolerated as a neoadjuvant chemotherapy for advanced gastric cancer. Toxicity is mild and the majority of the adverse events are of grade I or II, and no severe infections or mortality are involved (7). Therefore, this chemotherapy is well tolerated by the patients who received it. The FOLFOX regimen is one of the most frequently used chemotherapies for advanced gastric carcinoma in China (6,7). In 2007, Moon studied the FOLFOX regimen as a neoadjuvant chemotherapy for advanced gastric cancer and demonstrated that the clinical effectiveness was 58% with an R0 resection rate of 52% (23). In 2008, our preliminary report showed that neoadjuvant chemotherapy with FOLFOX was effective in gastric cancer and the toxicity levels were tolerable. Therefore, this treatment may improve the curative resection rate and survival of patients with locally advanced gastric cancer. The results further showed that the curative resection rates were 89.7 and 77.6% in the FOLFOX and surgery groups, respectively, and the mean survival was 20.6 and 19.9 months in the FOLFOX surgery groups, respectively (P=0.02) (6). Thus, neoadjuvant chemotherapy with FOLFOX is frequently selected prior to surgery to treat advanced stomach cancer. In the present study, 79.3% of the cases exhibited R0 resection and 19.5% developed tumour regression following chemotherapy. No severe adverse or grade IV events were recorded. Only four cases (4.6%) with grade III neutropenia and 18 cases (20.7%) with grade III nausea and vomiting were observed. For the FOLFOX regimen, the complete pathological response was low, in which only four cases (4.6%) developed complete tumour regression. At present, surgeons require a more effective regimen prior to surgery to increase R0 resection and prolong overall survival.

The British Medical Research Council performed the first well-established MAGIC trial for perioperative chemotherapy (4). In this trial, 503 patients randomly received perioperative chemotherapy with ECF and surgery or surgery alone. The five-year survival rates were 36 and 23% in the perioperative chemotherapy and surgery groups, respectively. Perioperative chemotherapy significantly improved the progression-free survival and the overall survival of patients with operable gastric and lower oesophageal adenocarcinomas. The results of this study have established perioperative chemotherapy as an alternative strategy to standard therapy for patients with resectable gastric cancer. In each group, 74, 14 and 11% of patients suffered from stomach cancer, lower oesophageal cancer and cancer of the oesophagogastric junction. However, the ECF regimen requires long hospitalisation rendering the application of this treatment difficult (4). Cunningham *et al*(24) studied capecitabine and oxaliplatin as alternative drugs to fluorouracil and cisplatin, respectively, for untreated advanced oesophagogastric cancer in the REAL-2 trial. The overall survival was longer with EOX than ECF, with the risk ratio of death at 0.80 in the EOX group (95% confidence interval, 0.66–0.97; P=0.02). The EOX regimen is more convenient than the FOLFOX for physicians and patients. The use of perioperative chemotherapy with the triplet regimen has been observed in Western countries. However, this method is not commonly used in China possibly due to the various conditions of gastric cancer recorded in China. For example, distal stomach cancer is more prevalent in Chinese patients than proximal stomach cancer. The triplet regimen may result in increased adverse events than the doublet regimen. In the present study, the effects and safety were compared between the various regimens. No lethal incidences resulting from preoperative chemotherapy were observed in the cases. FOLFOX was associated with a significantly lower grade III or IV nausea and vomiting compared with the EOX group (P=0.029). The symptoms of nausea and vomiting alleviated 3 or 4 days following administration of the chemotherapy agents. Grade III neutropenia was more frequent in the EOX group (11.5%) than the FOLFOX group (4.6%), but this difference was not significant (P=0.635). Bone marrow suppression was controlled by subcutaneously injecting granulocyte colony-stimulating factor. Grade I hand-foot syndrome was significantly more prevalent in the EOX group (34.6%) than in the FOLFOX group (16.1%). Other adverse events were not severe in the two groups prior to surgery. Therefore, the EOX regimen resulted in increased side effects compared with the FOLFOX regimen; however, these side effects were easily relieved. The two regimens were safe and tolerable and no major complications were observed in following surgery.

Neoadjuvant chemotherapy for gastric cancer should be further evaluated. However, the current approach used to evaluate the actual efficacy of chemotherapeutic agents is based on the clinical and pathological responses demonstrated in a previous study (15). Previous results have shown that the clinical response evaluation, based on World Health Organisation or RECIST criteria, is highly inaccurate in gastric cancer when using conventional staging modalities, including endoscopy, ultrasound, CT, magnetic resonance imaging and positron emission tomography (25). The type of pathological response and histological tumour regression following neoadjuvant therapy has been considered as a predictor of survival of patients with gastric adenocarcinoma. In our previous study, tumour regression was found in 22.2% (24/108) of patients, indicating that the patients with tumour regression exhibited higher overall survival than those without tumour regression (15). Patients with pathologically complete remission following neoadjuvant chemotherapy benefited from these treatment modalities (12,17). In cases with complete regression, 4 (4.6%) and 3 (11.5%) were observed in the FOLFOX and EOX groups, respectively. In addition, a higher number of patients (38.5%) developed tumour regression (TRG 3 and 4) in the EOX group than the FOLFOX group (19.5%). A significant difference was also observed between the two neoadjuvant chemotherapy regimens (P=0.047). Three cases with pathological complete regression received preoperative radiation therapy in the FOLFOX group, whereas the three other cases with complete regression did not receive this therapy in the EOX group.

Downstaging and increasing the radical resection rate are the primary objectives of preoperative chemotherapy in gastric cancer surgery. The results of the first stage III randomised clinical trial on neoadjuvant chemotherapy of gastric cancer suggested that neoadjuvant chemotherapy may significantly downstage tumours and increase the rate of R0 resection to approximately 10% (4,26,27). According to results of the CT scan with contrast, pretreatment clinical T and N stages were similar between the FOLFOX and EOX groups. Following neoadjuvant chemotherapy, the number of patients exhibiting metastatic lymph nodes was lower in the EOX group (57.7%) compared with the FOLFOX group (67.8%). A higher number of patients also exhibited deeper invasive cancer in the FOLFOX group (P=0.047). The EOX regimen increased the R0 resection rate compared with that of the FOLFOX regimen (84.6 vs. 79.3%, respectively). These observations indicate that EOX is potentially more effective than FOLFOX as a preoperative therapy.

In conclusion, the present retrospective study demonstrated that EOX may be more effective than FOLFOX as a preoperative chemotherapy for Chinese patients with advanced gastric cancer. However, FOLFOX may be the optimal regimen particularly for younger patients subjected to individual neoadjuvant chemotherapy. The side effects of the two regimens may be tolerated by administering supportive treatment. Although our results provided an alternative preoperative option for gastric surgeons, numerous difficulties remain unresolved. There were several limitations of this study, including a small sample size and the presentation of data from a single institution. Therefore, larger prospective clinical trials in several institutions should be undertaken in the future.

## Figures and Tables

**Table I:** Baseline demographic and clinical characteristics.

Variable	FOLFOX, n=87 (%)	EOX, n=26 (%)	t/χ^2^	P-value
Age	59.39±10.59	55.19±9.52	1.813	0.073
Gender				
Male	64 (73.6)	19 (73.1)	0.002	0.961
Female	23 (26.4)	7 (26.9)		
Tumour location			0.908	0.341
Upper body	14 (16.1)	2 (7.7)		
Middle body	21 (24.1)	7 (26.9)		
Lower body	47 (54)	14 (53.8)		
Diffuse type	5 (5.7)	3 (11.5)		
Tumour differentiation			2.009	0.571
Differentiated	29 (33.3)	8 (30.8)		
Undifferentiated	58 (66.7)	18 (69.2)		
Clinical T classification			0.444	0.801
T2	9 (10.3)	2 (7.7)		
T3	44 (50.6)	15 (57.7)		
T4	34 (39.1)	9 (34.6)		
Clinical N classification			0.209	0.647
N^−^	31 (35.6)	8 (30.8)		
N^+^	56 (64.4)	18 (69.2)		
Tumour size, cm				
<6	53 (60.9)	16 (61.5)		
>6	34 (39.1)	10 (38.5)		
Chemotherapy cycles	3.86±1.85	3.80±1.36	0.139	0.890

**Table II:** Analysis of efficacy.

Variable	FOLFOX, n=87 (%)	EOX, n=26 (%)	t/χ^2^	P-value
Surgical procedure				
Radical operation, R0	69 (79.3)	22 (84.6)	0.359	0.549
Palliative operation, R1	18 (20.7)	4 (15.4)		
Pathological T classification			1.382	0.047
pT0	5 (5.7)	3 (11.5)		
pT1	8 (9.2)	4 (15.4)		
pT2	8 (9.2)	3 (11.5)		
pT3/pT4	66 (75.9)	16 (61.5)		
Pathological N classification			20.983	0.000
N^−^	28 (32.2)	11 (42.3)		
N^+^	59 (67.8)	15 (57.7)		
TRG			6.641	0.156
0	10 (11.5)	5 (19.2)		
1	28 (32.2)	4 (15.4)		
2	32 (36.8)	7 (26.9)		
3	13 (14.9)	7 (26.9)		
4	4 (4.6)	3 (11.5)		
Pathological response			3.941	0.047
TRG 0–2	70 (80.5)	16 (61.5)		
TRG 3,4	17 (19.5)	10 (38.5)		

TRG, tumour regression grade.

**Table III:** Specific treatment-related adverse events.

Adverse event, grade	FOLFOX, n=87 (%)	EOX, n=26 (%)	t/χ^2^	P-value
Neutropenia				
0	20 (23)	6 (23.1)	1.708	0.635
I	25 (28.7)	7 (26.9)		
II	38 (43.7)	10 (38.5)		
III	4 (4.6)	3 (11.5)		
Anemia			0.099	0.952
0	41 (47.1)	13 (50)		
I	27 (31)	8 (30.8)		
II	19 (21.8)	5 (19.2)		
Thrombocytopenia			0.326	0.850
0	77 (88.5)	23 (88.5)		
I	9 (10.3)	3 (11.5)		
II	1 (1.1)	0 (0)		
Nausea and vomiting			10.812	0.029
0	1 (1.1)	0 (0)		
I	15 (17.2)	5 (19.2)		
II	53 (60.9)	13 (50)		
III	18 (20.7)	5 (19.2)		
IV	0 (0)	3 (11.5)		
Peripheral neuropathy			1.601	0.449
0	53 (60.9)	15 (57.7)		
I	25 (28.7)	10 (38.5)		
II	9 (10.3)	1 (3.8)		
Hand-foot syndrome			4.237	0.040
0	73 (83.9)	17 (65.4)		
I	14 (16.1)	9 (34.6)		
ALT/AST increase			0.001	0.980
0	60 (69)	18 (69.2)		
I	27 (31)	8 (30.8)		

ALT, alanine transferase; AST, aspartate transferase.
